# The Effect of MSTN Mutation on Bile Acid Metabolism and Lipid Metabolism in Cattle

**DOI:** 10.3390/metabo13070836

**Published:** 2023-07-11

**Authors:** Di Wu, Song Wang, Chao Hai, Linfeng Wang, Dongchao Pei, Chunling Bai, Guanghua Su, Xuefei Liu, Yuefang Zhao, Zhonghua Liu, Lei Yang, Guangpeng Li

**Affiliations:** 1 State Key Laboratory of Reproductive Regulation and Breeding of Grassland Livestock, College of Life Science, Inner Mongolia University, Hohhot 010021, China; 2 College of Life Science, Northeast Agricultural University, Harbin 150030, China

**Keywords:** MSTN, bile acids, lipid metabolism, liver, enterohepatic circulation

## Abstract

Myostatin (MSTN) is a negative regulator of skeletal muscle genesis during development. MSTN mutation leads to increased lean meat production and reduced fat deposition in livestock. However, the mechanism by which MSTN promotes myogenesis by regulating metabolism is not clear. In this study, we compared the metabolomics of the livers of wild-type (WT) and MSTN mutation cattle (MT), and found changes in the content and proportion of fatty acids and bile acids in MT cattle. The differential metabolites were enriched in sterol synthesis and primary bile acid synthesis. We further analyzed the expression of genes involved in the regulation of lipid and bile acid metabolism, and found that the loss of MSTN may alter lipid synthesis and bile acid metabolism. This study provides new basic data for MSTN mutations in beef cattle breeding.

## 1. Introduction

Myostatin (MSTN) is a protein that belongs to the TGF-β superfamily and exerts a pivotal function in regulating muscle growth and development in animals [1]. MSTN deactivation by genetic deletion or natural mutation causes muscle hypertrophy, which is widely reported in a variety of mammals [2,3,4,5]. In cattle breeding, MSTN has been a major subject research topic as a potential target for improving growth and production traits [6,7,8]. Using biological breeding technology, we conducted site-specific gene editing inactivation of MSTN by CRISPR/Cas9, effectively reduced the expression of MSTN, produced the purebred MSTN knockout Luxi cattle, which stably inherited the double muscle phenotype caused by MSTN deletion, forming a new breeding strain [9]. In previous studies, we have proved that the health performance and reproductive capacity of cattle after MSTN knockout are basically the same as wild-type cattle, with corresponding increases in growth rate, carcass rate and lean meat yield [9]. MSTN also plays a crucial role in glucose metabolism and energy metabolism in vivo. Further, our studies on the skeletal muscles of MSTN mutants showed that glycolysis, glycogen metabolism and adipose β-oxidation processes were significantly altered [10,11,12]. It has been reported that MSTN can promote oxidative catabolism of white fat in the body by regulating glucose metabolism or changing hormone levels [13]. Although MSTN has the potential to reduce fat deposition in animals, our understanding of how MSTN regulates lipid metabolism is still limited. The liver is the main lipid metabolizing organ in human body and plays a crucial role in the synthesis and degradation of lipids [14]. In this case, the liver is also the main site of bile acid synthesis, a small molecule that plays a key role in the digestion and absorption of dietary fats and is involved in the regulation of cholesterol and other lipids in the liver [15]. The primary bile acids synthesized in the liver need to combine with amino acid groups (especially taurine and glycine), glycosides, sulfate groups and other functional groups in the presence of catalytic proteins to form conjugated bile acids, which are then transported by the bile salt outlet pump and stored in the gallbladder, and then stimulated by ingestion into the intestine with bile [16].

Bile acids entering the small intestine form secondary bile acids, which facilitate the digestion and absorption of lipids through multiple steps such as oxidation, differential isomerization, 7-dehydroxylation and recombination [17,18]. Finally, bile acids are passively diffused and actively transported to the end of ileum, and about 90% bile acids are absorbed into the liver through the hepatic portal vein to participate in a new round of digestion and absorption [19,20]. The process of bile acids synthesis, transport, secretion and reabsorption is known as the bile acids enterohepatic circulation, which is the most important part of bile acid metabolism [21,22]. It has been reported that changes in the composition and content of bile acids directly affect the digestion and absorption of lipids to a large extent, and thus affect lipid metabolism [23]. Dietary fats are broken down by bile acids into glycerol and fatty acids, which are then transported by plasma to the liver for synthesis and decomposition [24]. The liver is highly related to fatty acid metabolism and bile acids metabolism.

In our previous study, we suggested that MSTN mutations might increase primary bile acid synthesis and improve serum lipid concentrations [25]. However, there are few studies on the effect of MSTN on bile acid metabolism, and the understanding of its mechanism is very limited. Therefore, the aim of this study was to explain the effect of MSTN mutation on liver lipid metabolism and bile acids by measuring and analyzing the expression of bile acid metabolites and related genes in liver and intestinal circulation. This study also provides potential ideas for further exploring the possible problems and mechanisms of MSTN in the breeding process of beef cattle.

## 2. Materials and Methods

### 2.1. Experimental Animals and Sample Collection

Animal care and experimental protocols were approved by the Animal Ethics Committee of Inner Mongolia University (No. IMU-CATTLE-2022-073) and were carried out in accordance with the committee’s guidelines for animal research. As mentioned in our previous report, we used CRISPR/Cas9 technology and somatic cell nuclear transplantation to produce MSTN knockout Luxi cattle (MSTN^−/−^ Luxi cattle ♂, *Bos taurus*), and used lineage progenitor hybridization with wild-type female Luxi cattle to successfully produce a population of MSTN^+/−^ Luxi cattle [9,26]. In this study, a total of twelve 2-year-old steers were used, with six MSTN^+/−^ Luxi cattle (MT) as the experimental group and six wild-type Luxi cattle (WT) as the control group. The wild-type cattle were not genetically edited and all are purebred Luxi cattle.

All experimental animals were housed in Hohhot, Inner Mongolia, China (111°85′ E, 40°55′ N, 1040 m above sea level). Each barn was equipped with a temperature-controlled (15 °C) automatic watering system, and all cattle were allowed to drink freely. The total mixed ration (TMR) consists of 68% silage, 12% hay and 20% grain feed. Each barn was a separate compartment containing approximately 30 m^2^ of indoor area and approximately 300 m^2^ of outdoor area shared by each group of cattle (*n* = 6), with all cattle being allowed unrestricted access to both indoor and outdoor areas. Cattle were fed until 24 months of age for slaughter, and fasted for 24 h before slaughter and allowed to drink freely. Slaughter was carried out in the morning, and all cattle were slaughtered by bloodletting. The slaughtering process followed national standard operating procedures (GB/T 19477-2018 [27], Cattle slaughter, Beijing, China). Liver tissue was collected within 30 min following slaughter, along with semitendinosus tissue from the rump of the cattle. These samples were subsequently divided into smaller portions and rapidly submerged in liquid nitrogen for freezing. Afterward, the frozen specimens were transferred to a −80 °C freezer for storage until needed for further analysis. The ileal contents were also collected within 30 min after slaughter and these contents were taken from the middle segment of the cattle ilea. The contents were packed into clean collection tubes, tightly capped, and then first immersed in liquid nitrogen and frozen, then transferred to a −80 °C refrigerator until further analysis.

### 2.2. Fatty Acid Content Analysis in Liver

All samples were detected on a gas chromatograph–mass spectrometer (GC-2010 GC–MS, Shimadzu, Kyoto, Japan). The frozen liver tissues were weighed at 0.1 g on a balance, then we added 3 mL of chloroform–methanol solution (2:1), mixed by grinding and crushing and stored at 25 °C for 4 h. The supernatant was removed by centrifugation at 10,000× *g* for 5 min and the supernatant was dried by purging in nitrogen. The precipitate was dissolved in 500 μL of n-hexane, then we added 1 mL of saturated KOH–methanol solution, left it standing at 25 °C for 4 h, and centrifuged for 10 min at 1500× *g*, and then the sample was placed in the injection vial. The GC column (HP-88, film thickness 0.20 μm, length 100 m, inner diameter 0.25 mm, injection volume 1 μL, Agilent, Palo Alto, USA, 112-88A7E) chamber temperature was 60 °C, the injection port temperature was 220 °C, the injection method was split. The protocol was to reach 60 °C, hold for 1 min, ramp up to 140 °C and hold for 10 min at a rate of 40 °C/min, then ramp up to 240 °C at a rate of 4 °C/min and hold for 15 min. Agilent Masshunter analysis software (version 8.0) was used for instrument control, data collection, processing and analysis.

### 2.3. Targeted Metabolomic Analysis of Bile Acids in Liver Tissues and Ileal Contents

All samples were detected on an ultra-performance liquid chromatography–mass spectrometer (1290-6470 UPLC-MS/MS. Agilent, Palo Alto, USA). Bile acids were extracted using the previously published procedure with some minor modifications [28]. The thawed liver sample was mixed with methanol at a concentration of 67% and centrifuged at 14,000× *g* for 10 min at 4 °C to remove the protein. The supernatant was dried under nitrogen purge and redissolved with 100 µL of 50% methanol solution containing 0.005% formic acid for UPLC-MS/MS analysis; extraction of homogenized samples of ileal contents was performed by adding 10 volumes (*v*/*w*) of pre-cooled methanol solution and subsequently performing sonication for 5 min, followed by centrifugation of the mixture at 14,000× *g* for 10 min at 4 °C. After collecting the supernatant, the extraction was repeated by adding another 10 volumes of methanol. The supernatants obtained from the two extractions were combined, further diluted 20-fold and mixed with 100 ng/mL IS solution (50% aqueous methanol) in equal amounts.

1 μL of the extraction was injected using a Kinetex Core-Shell 2.6 µm C18 column (100 × 2.1 mm, 2.6µm, Phenomenex, Torrance, CA, USA) using a flow rate of 0.6 mL/min at 45 °C during a 15 min gradient; gradient elution conditions were set as follows: 0~2 min from 23% B to 33% B, 2~6 min from 33% B to 34% B, 6~11 min from 34% B to 70% B, 11~11.01 min from 70% B to 95% B, 11.01~15 min 95% B. We used solvent A, water containing 0.005% formic acid, and solvent B, acetonitrile containing 0.005% formic acid. The pressure of the nebulizer was 20 psi, the sheath gas temperature was 350 °C with a flow rate of 10 L/min, the dry gas temperature was 350 °C with a flow rate of 10 L/min, the capillary was set at 3500 V. Multiple reaction monitoring (MRM) has been used for quantification of screening fragment ions. More details about MRM such as Quantifier MRM transitions and Retention time (min) are provided in the Supplementary Materials, as detailed in Table S5. Agilent Masshunter analysis software (version 8.0) was used for instrument control, data collection, processing and analysis.

### 2.4. Non-Targeted Lipid Metabolic Analysis of the Liver

We weighed a 1 g tissue sample, added 3 mL methanol and 0.64 mL ultrapure water to it, and ground it at a low temperature (0 °C). The samples were centrifuged at 4 °C for 10 min at 12,000× *g*. Extracted bottom compounds (containing lipophilic compounds) were transferred to a separate injection vial for LC–MS (LC-Bio, Hangzhou, China) analysis. All samples were acquired by the LC–MS system following the manufacturer’s instructions. Firstly, all chromatographic separations were performed using an ultra-performance liquid chromatography (UPLC) system (SCIEX, Warrington, UK). Metabolite separation was performed using an ACQUITY UPLC HSS T3 column (100 mm × 2.1 mm, 1.8 m, Waters, Manchester, UK) to analyze liver tissue samples. The column oven was maintained at 35 °C. The flow rate was 0.4 mL/min and the mobile phase consisted of solvent A (water, 0.1% formic acid) and solvent B (acetonitrile, 0.1% formic acid). Gradient elution conditions were set as follows: 0~0.5 min, 5% B; 0.5~7 min, 5% to 100% B; 7~8 min, 100% B; 8~8.1 min, 100% to 5% B; 8.1~10 min, 5% B. The injection volume for each sample was 4 µL.

Raw data files were converted using XCMS, CAMERA and metaX toolboxes [29,30,31]. Metabolites were identified by KEGG and HMDB website (http://www.hmdb.ca/, accessed on 10 December 2022) metabolic databases, and HMDB IDs were generated accordingly. In the process of screening out differentially metabolic features, univariate analysis of metabolites was performed, mainly to calculate the fold change (FC) of the features of metabolites in the WT and MT groups; *t*-testing was performed to detect differences in metabolite features between the two groups, and *p*-values were adjusted for multiple testing using FDR (Benjamini-Hochberg); supervised partial least squares discriminant analysis (PLS-DA) was performed by metaX to distinguish different variables, and variable importance in projection (VIP) scores were calculated. The differential metabolites met both: 1. FC ≥ 2 or FC ≤ 0.5; 2. *p*-value < 0.05; 3. VIP ≥ 1. The differential metabolite features were normalized by Z-score to the intensity values of each metabolite for different samples and then presented in a heatmap by the package pheatmap of R (version 4.2.1). Finally, enrichment analysis of differential metabolites was performed through the MetaboAnalyst 5.0 website (https://www.metaboanalyst.ca/MetaboAnalyst/home.xhtml, accessed on 17 December 2022) and the results are presented in the bar plot.

### 2.5. Real-Time PCR

Total RNA from liver tissues was extracted by using the RNAiso Plus kit (Takara, Shiga, Japan, 9108) according to the manufacturer’s instructions. cDNA was extracted by using the PrimeScript RT kit and gDNA Eraser (Perfect Real Time) (Takara, Shiga, Japan, RR047A). We used an ABI 7500 real-time PCR instrument (Applied Biosystems, Foster City, CA, USA) and SYBR Green reagent (Takara, Shiga, Japan, RR820A) for amplification reactions.

PCR amplification was performed under the following conditions: preheating at 95 °C for 30 s, then a cycling process of 95 °C for 5 s and 60 °C for 34 s, with 40 cycles, followed by a melting curve plotting and analysis phase for about 30 min. The fold change in gene expression was determined using the comparative threshold cycling (2^−∆∆Ct^) method and GAPDH was used as an internal reference gene. The primers used are in Table S1 of the Supplementary Materials.

### 2.6. Western Blot

The total protein was extracted from the liver and the muscle of the cattle by homogenization in ice-cold radio immunoprecipitation assay (RIPA) buffer. The liver tissue lysates and the muscle tissue lysates were then centrifuged at 4 °C for 30 min at 8000× *g*. The protein concentration was determined by BCA assay (Thermo, Waltham, MA, USA, 23225). The supernatant was electrophoresed in 10% SDS-polyacrylamide gel and transferred onto a polyvinylidene difluoride membrane by electroblotting. The membrane was blocked in 5% non-fat milk in Tris-buffered saline with 0.1% Tween-20 (TBST) blocking solution at room temperature for 1 h, and incubated with anti-MSTN (Proteintech, Wuhan, China, 19142-1-AP) and anti-α Tubulin (Proteintech, Wuhan, China, 11224-1-AP) in TBST containing 0.5% non-fat milk at 4 °C overnight. The membranes were then incubated for 1 h with horseradish peroxidase-conjugated goat anti-mouse and anti-rabbit secondary antibodies (1:10,000) at room temperature, followed by detection using the chemiluminescence labeling detection reagent ECL Plus (Thermo, Waltham, MA, USA, 32209). α Tubulin was used as the loading control.

### 2.7. Statistical Analysis

The data are shown as the mean ± SD (*n* = 6). Statistical analyses were performed using GraphPad Prism 9.5.0 software. Data were checked for normal distribution by the Shapiro–Wilk test and homogeneity of variance by Levene’s test to ensure the data conformed to the Student’s *t*-test. Student’s *t*-test was used to calculate the *p*-value: *p* < 0.05 was considered statistically significant.

## 3. Results

### 3.1. Analysis of Fatty Acid Content and Composition in Livers of the Cattle

To examine the expression of MSTN in cattle, we first analyzed the RNA expression level of MSTN by RT-qPCR and the protein expression level of MSTN by Western blot. The results are shown in Figure S1A,B, respectively. We observed a significant down-regulation of MSTN expression in MT cattle compared to WT cattle in both muscle and liver tissues. These results are consistent with our expected results using WT cattle and MSTN^+/−^ MT cattle.

**Figure 1:**
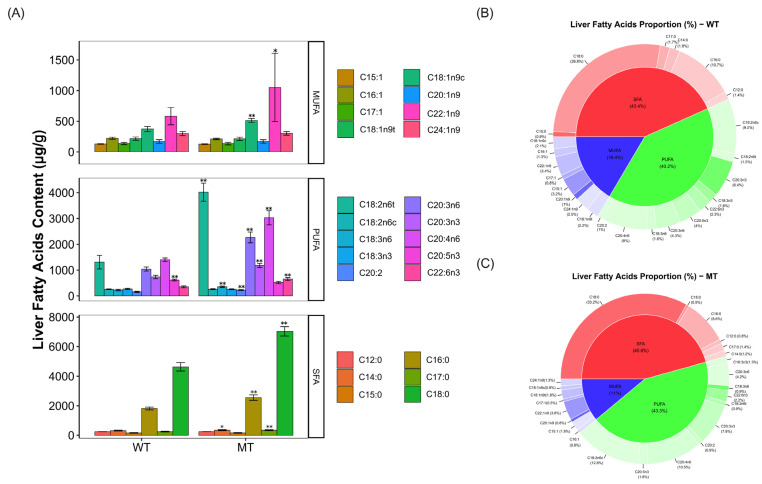
Analyses of the content and proportion of fatty acids in the livers of WT and MT cattle. (**A**) Fatty acids content in the livers of WT and MT cattle. (**B**,**C**) Proportion of fatty acids in the livers of WT and MT cattle. WT, wild type; MT, MSTN mutation. Abbreviations: C15:1, cis-10-Pentadecenoic acid; C16:1, Palmitoleic acid; C17:1, cis-10-Heptadecenoic acid; C18:1n9t, Elaidic acid; C18:1n9c, Oleic acid; C20:1n9, cis-11-Eicosenoic acid; C22:1n9, Erucic acid; C24:1n9, Nervonic acid; C18:2n6t, Linolelaidic acid; C18:2n6c, Linoleic acid; C18:3n3, a-Linolenic acid; C18:3n6, y-Linolenic acid; C20:2, cis-11.14-Eicosadienoic acid; C20:3n3, Eicosatrienoic acid triesters; C20:3n6, Eicosatrienoic acid methyl ester; C20:4n6, arachidonic acid; C20:5n3, cis-5,8,11,14,17-Eicosapentaenoic acid; C22:6n3, Docosahexaenoic acid; C12:0, Lauric acid; C14:0, Myristic acid; C15:0, Pentadecanoic acid; C16:0, Palmitic acid; C17:0, Heptadecanoic acid; C18:0, Stearic acid. Unit: (μg/g); data presented as mean ± SD; compared with the control group, * *p* < 0.05, ** *p* < 0.01; Student’s *t*-test was used to calculate the *p*-values. We used *n* = 6 cattle per group.

Since the liver is one of the most important organs for fatty acid metabolism, we detected fatty acids in liver tissues by GC–MS and analyzed the effect of MSTN mutation on fatty acid content changes. A total of 24 fatty acids were detected in the livers of both MT and WT cattle. In the polyunsaturated fatty acids, the contents of C18:2n6t (MT vs. WT, 4020.29 ± 349.5 μg/g vs. 1309.73 ± 263.07 μg/g, *p* = 0.00595), C18:3n6 (MT vs. WT, 355.3 ± 27.01 μg/g vs. 226.68 ± 32.01 μg/g, *p* = 0.00155), C20:2 (MT vs. WT, 226.81 ± 18.55 μg/g vs. 158.73 ± 21.6 μg/g, *p* = 0.00194), C20:3n6 (MT vs. WT, 2266.59 ± 208.16 μg/g vs. 1040.6 ± 78.24 μg/g, *p* = 0.0036), C20:3n3 (MT vs WT, 1185.83 ± 78.55 μg/g vs. 734.61 ± 66.55 μg/g, *p* = 0.0050), C20:4n6 (MT vs WT, 3028.52 ± 272.3 μg/g vs. 1403.78 ± 66.49 μg/g, *p* = 0.0054) and C22:6n3 (MT vs. WT, 655.91 ± 55.33 μg/g vs. 353.68 ± 35.06 μg/g, *p* = 0.0011) were significantly increased in MT cattle (Figure 1A; Table S2).

In the saturated fatty acids, the contents of C14:0 (MT vs. WT, 345.61 ± 33.97 μg/g vs. 316.49 ± 26.69 μg/g, *p* = 0.01539), C16:0 (MT vs. WT, 2551.17 ± 182.5 μg/g vs. 1813.83 ± 97.09 μg/g, *p* = 0.0035), C17:0 (MT vs. WT, 344.96 ± 28.27 μg/g vs. 246.08 ± 32.64 μg/g, *p* = 0.00484) and C18:0 (MT vs. WT, 7627.22 ± 318.3 μg/g vs. 4635.14 ± 280.61 μg/g, *p* = 0.0033) were significantly elevated in MT cattle (Figure 1A; Table S2).

In terms of liver fatty acid proportion, compared to WT cattle, the percentage of polyunsaturated fatty acids and the percentage of saturated fatty acids in the liver of MT cattle increased by 3.1% (MT vs. WT, 43.3% vs. 40.2%) and 2.4% (MT vs. WT, 45.8% vs. 43.4%), respectively (Figure 1B,C). In contrast, the percentage of monounsaturated fatty acids decreased by 5.4% (MT vs. WT, 11% vs. 16.4%). Among them, C18:0 increased by 6.4% (MT vs. WT, 33.2% vs. 26.8%), C18:2n6c by 3.3% (MT vs. WT, 12.8% vs. 9.5%) and C20:4n6 by 2.5% (MT vs. WT, 10.5% vs. 8%) in MT cattle liver compared to WT cattle (Figure 1B,C). These results suggest that the MSTN mutation alters the content and proportion of fatty acids in the livers of cattle.

### 3.2. Analysis of Bile Acid Content and Proportion in Liver and Ileal Contents of the Cattle

**Figure 2:**
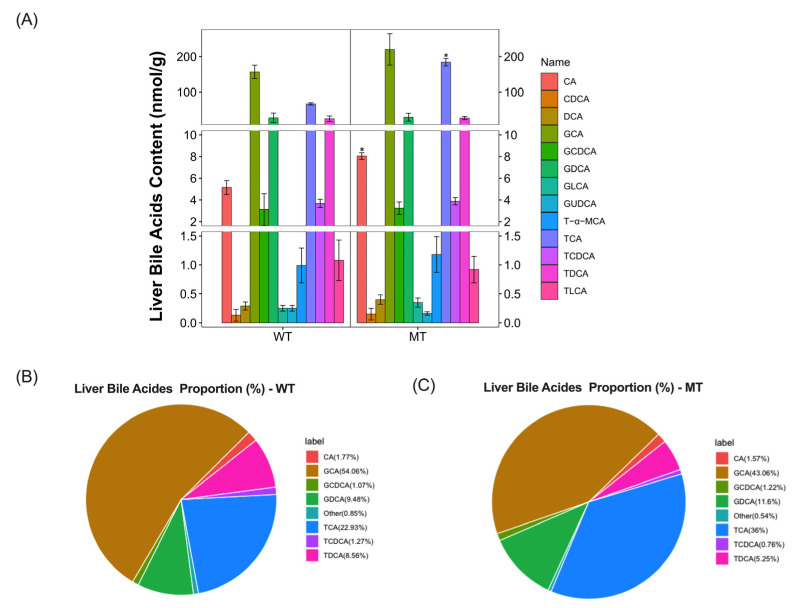
Bile-acid-targeted metabolomic analysis of the content and composition of bile acids in livers of WT and MT cattle. (**A**) Bile acids content in the livers of WT and MT cattle. (**B**,**C**) Composition of bile acids in the livers of WT and MT cattle. WT, wild type; MT, MSTN mutation. Abbreviations: CA, Cholic acid; CDCA, Chenodeoxycholic acid; DCA, Deoxycholic acid; GCA, Glycocholic acid; GCDCA, Glycochenodeoxycholic acid; GDCA, Glycodeoxycholic acid; GLCA, Glycolithocholic acid; GUDCA, Glycoursodeoxycholic acid; LCA, Lithocholic acid; T-α-MCA, Tauro-α-muricholic acid; TCA, Taurocholic acids; TCDCA, Taurochenodeoxycholic acid; TDCA, Tauroursodeoxycholic acid; TLCA, Taurolithocholic acid. Unit: (nmol/g); Data presented as mean ± SD; compared with the control group, * *p* < 0.05; Student’s *t*-test was used to calculate the *p*-values. We used *n* = 6 cattle per group.

To further characterize the differences in bile acids content and proportion in the livers and ilea of cattle after MSTN mutation, we used a UPLC-MS/MS based on a bile-acid-targeted metabolomic to measure bile acids content and proportion in the livers of the cattle. We detected 14 bile acids in the livers of WT and MT cattle (Figure 2A; Table S3). Compared with WT cattle, MT cattle livers had increased bile acid content, significantly increased CA (MT vs. WT, 8.05 ± 0.31 nmol/g vs. 5.15 ± 0.63 nmol/g, *p* = 0.02272) and TCA (MT vs. WT, 184.08 ± 10.59 nmol/g vs. 66.55 ± 3.35 nmol/g, *p* = 0.03396), and no significant difference in GCA (MT vs. WT, 220.2 ± 44.39 nmol/g vs. 156.91 ± 18.54 nmol/g, *p* = 0.82124).

In terms of bile acid proportion, CA (MT vs WT, 1.57% vs. 1.77%) and GCA (MT vs. WT, 43.06% vs. 54.06%) decreased relatively after MSTN mutation, while the proportion of TCA increased by 13.07% (MT vs. WT, 36% vs. 22.93%), and the proportion of taurine-conjugated bile acids increased. Similarly, the proportion of conjugated bile acids involving glycine were reduced (Figure 2B,C).

**Figure 3:**
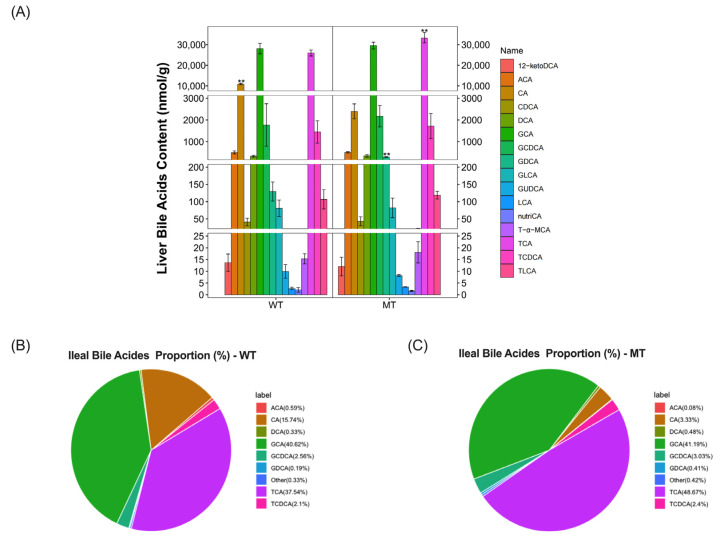
Bile-acids-target metabolomics analysis of the content and composition of bile acids in the ileal digesta of WT and MT cattle. (**A**) Bile acids content in the ileal contents of WT and MT cattle. (**B**,**C**) Composition of bile acids in the ileal digesta of WT and MT cattle. WT, wild type; MT, MSTN mutation. Abbreviations: 12-ketoDCA, 12-ketodeoxycholic acid; ACA, Acetylcholic acid; CA, Cholic acid; CDCA, Chenodeoxycholic acid; DCA, Deoxycholic acid; GCA, Glycocholic acid; GCDCA, Glycochenodeoxycholic acid; GDCA, Glycodeoxycholic acid; GLCA, Glycolithocholic acid; GUDCA, Glycoursodeoxycholic acid; LCA, Lithocholic acid; nutriCA, Nutritional cholic acid; T-α-MCA, Tauro-α-muricholic acid; TCA, Taurocholic acids; TCDCA, Taurochenodeoxycholic acid; TLCA, Taurolithocholic acid. Unit: (nmol/g); Data presented as mean ± SD; compared with the control group, ** *p* < 0.01, Student’s *t*-test was used to calculate the *p*-values. We used *n* = 6 cattle per group.

Subsequently, we performed targeted metabolite analysis of the ileal contents by UPLC-MS/MS. We identified 16 variants of bile acids in the ilea of both WT and MT cattle (Figure 3A; Table S4). Compared to WT cattle, the ileal contents of MT cattle had a highly significant increase in TCA (MT vs. WT, 34,949.68 ± 2490.93 nmol/g vs. 25,967.26 ± 1452.57 nmol/g, *p* = 0.00024), whereas the content of GCA (MT vs. WT, 29,578.4 ± 1645.7 nmol/g vs. 28,097.2 ± 2582.51 nmol/g, *p* = 0.82124) was not significantly different between the two group of cattle. It is worth noting that that CA (MT vs. WT, 2394.1 ± 343.04 nmol/g vs 10,886.86 ± 427.9 nmol/g, *p* = 0.00136) in the ilea of MT cattle was significantly reduced. The ileal bile acids proportion was also changed in MT cattle. Compared with WT cattle, the proportion of CA decreased by 12.41% (MT vs. WT, 3.33% vs. 15.74%), and the proportion of TCA (MT vs. WT, 48.67% vs. 37.54%) increased by 11.13% (Figure 3B,C). The results suggested that the content and proportion of bile acids in the liver and ilea of MT cattle were changed.

### 3.3. Non-Targeted Analysis of Liver Lipid Metabolism

**Figure 4:**
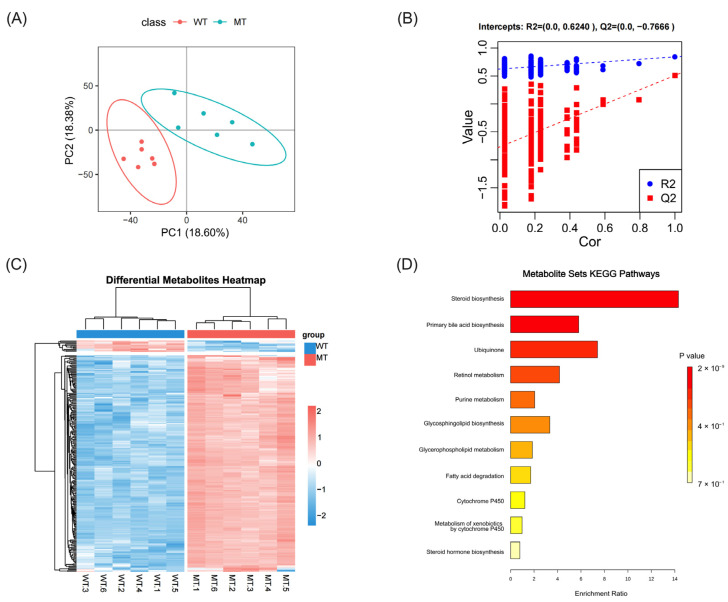
Non-targeted lipid metabolomic analysis of liver tissues in WT and MT cattle. (**A**) PLS-DA plot of the WT and MT groups; (**B**) permutation plot of the WT and MT groups. (**C**) Differential metabolite heat map of WT and MT cattle. (**D**) Enrichment pathways for differential metabolite sets in WT and MT cattle; WT, wild type; MT, MSTN mutation. We used *n* = 6 cattle per group.

To further investigate the changes in lipid metabolism in the livers of MT cattle, we detected metabolites in the liver by UPLC-MS/MS using non-targeted lipid metabolomics. We analyzed and identified metabolites by comparing them with the HMDB database; a total of 10,660 metabolites were obtained. Partial least squares discriminant analysis (PLS-DA) was further used to model the metabolite differences between MT and WT cattle. As shown in Figure 4A, each point in the plot represents one sample, and the results show a clear separation of the two groups. The PLS-DA model parameters R2 and Q2 were 0.841 and 0.509, indicating good applicability and predictive ability of the model. In addition, as shown in Figure 4B, in the permutation test, the intercept of the regression line of Q2 on the vertical axis is less than 0, indicating that the model was not overfitted and the differential metabolite analysis was more accurate. These results suggest that there are significant biochemical differences between MT and WT cattle. Based on the results of PLS-DA, the screening criteria for differential metabolites needed to satisfy both FC ≥ 2 and ≤0.5 are VIP ≥ 1 and *p* < 0.05. In total, there were 533 different metabolites between MT and WT cattle, of which 477 were up-regulated metabolites and 60 were down-regulated metabolites (Figure 4C, Table S6).

To further understand the biological function of the differential metabolites, KEGG functional annotation and pathway enrichment analysis of the differential metabolites were performed. Through the MetaboAnalyst 5.0 website (https://www.metaboanalyst.ca/MetaboAnalyst/home.xhtml, accessed on 17 December 2022) pathway analysis function, the HMDB IDs of differential metabolites were used for pathway enrichment analysis. The results showed that 11 metabolic pathways were found from MT and WT cattle. Of these pathways, only two were enriched, namely for steroid biosynthesis (*p* = 0.00000000216) and primary bile acid synthesis (*p* = 0.00411), respectively. The two pathways are closely related to bile acid metabolism and fatty acid metabolism. These results suggest that the lipid metabolism in the liver of cattle is altered after MSTN mutation.

### 3.4. Analysis of Gene Expression Related to Bile Acid Metabolism and Lipid Metabolism

**Figure 5:**
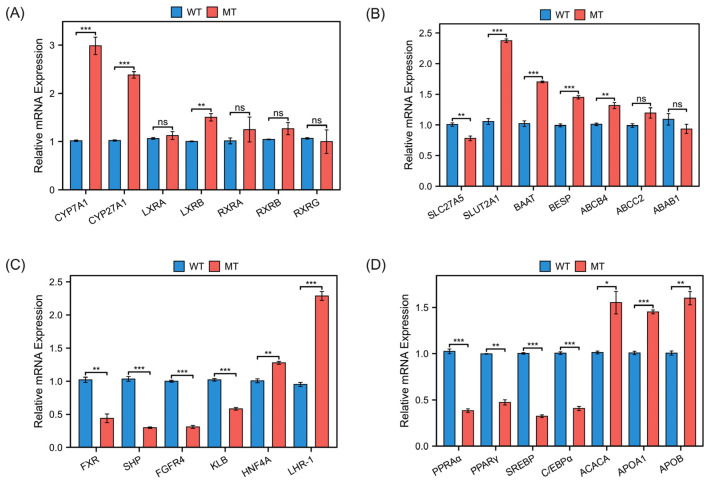
Relative expression analysis of genes related to bile acid metabolism and lipid metabolism in the livers of WT and MT cattle. (**A**) Relative expression of genes involved in bile acid synthesis in the livers of WT and MT cattle. (**B**) Relative expression of genes involved in bile acid maturation and secretion in the livers of WT and MT cattle. (**C**) Relative expression of genes involved in the regulation of bile acid metabolism and bile acid transport proteins in the livers of WT and MT cattle. (**D**) Relative expression of genes involved in lipid metabolism in the livers of WT and MT cattle. WT, wild type; MT, MSTN mutation. Data presented as mean ± SD. Compared with the control group, ns *p* ≥ 0.05, * *p* < 0.05, ** *p* < 0.01, *** *p* < 0.001; Student’s *t*-test was used to calculate the *p*-values. We used *n* = 6 cattle per group.

To obtain evidence that MSTN mutations cause changes in bile acid and lipid metabolism in cattle livers, we analyzed the expressions of genes related to bile acid and lipid metabolism. The expressions of cholesterol 7 alpha-hydroxylase (CYP7A1) and sterol 27-hydroxylase (CYP27A1), the major genes involved in bile acid synthesis and components of the cytochrome P450 family, were significantly up-regulated in MSTN cattle. The expression of liver X receptor beta (LXRB), which is also involved in bile acids synthesis, was significantly up-regulated (Figure 5A). After synthesis of the primary bile acids, conjugated bile acids are formed by adding sulfate groups, hydroxyl groups, sugar groups or amino acid groups through catalytic enzymes, thereafter to be transported and secreted. During this process, the expressions of sulfotransferase family 2A member 1 (SLUT2A1) and bile acid-CoA: amino acid N-acyltransferase (BAAT) were significantly up-regulated; meanwhile, the expressions of bile salt export pump (BESP) and ATP binding cassette subfamily B member 4 (ABCB4), which are involved in bile acids secretion and exocytosis, were significantly increased, while the expression of solute carrier family 27 member 5 (SLC27A5) was significantly decreased (Figure 5B).

We also examined the expression of genes involved in the regulation of bile acid metabolism and bile acid transport. Bile acid metabolism has a complex regulatory network, in which the farnesoid X receptor (FXR) inhibits bile acid synthesis by activating the small heterodimer partner (SHP). In addition, fibroblast growth factor receptor 4 (FGFR4) and beta-klotho (KLB) form heterotrimeric complexes that inhibit CYP7A1 activity and inhibit bile acid synthesis. According to our results, the expressions of bile acid regulatory factors FXR, SHP, and FGFR4/KLB were significantly decreased in the livers of MT cattle compared with WT cattle, while the expressions of hepatocyte nuclear factor-4α (HNF4α) and liver receptor homolog-1 (LHR-1) were significantly increased in MT cattle (Figure 5C). These results suggest that MSTN mutations promote the synthesis and secretion of bile acids, and cause changes in bile acid metabolism.

Moreover, the expressions of genes associated with lipid metabolism, such as peroxisome-proliferator-activated receptors PPARα, PPARγ, CCAAT-enhancer-binding proteins C/EBPα and sterol regulatory element-binding proteins (SREBP) were significantly down-regulated in MT cattle. The expressions of genes involved in lipid oxidation, fatty acids and lipid transport such as apolipoprotein (APO) A1, APOB and Acetyl-CoA carboxylase (ACACA) were significantly up-regulated in MT cattle (Figure 5D). These results suggest that MSTN mutations lead to altered lipid metabolism by reducing lipid synthesis capacity and enhancing fatty acid transport and metabolism.

## 4. Discussion

The MSTN gene is widely recognized for its high conservation across animal species and its role in regulating muscle development. MSTN has garnered significant interest in medical and biological breeding applications, particularly in the treatment of muscular dystrophy and livestock breeding [32,33,34]. Studies on naturally mutated cattle have shown a common phenotype characterized by a reduction in voluntary feed intake but a significant increase in feed efficiency, without significant reductions in maintenance requirements [35,36]. It has also been reported that an increase in meat yield and carcass weight, along with a decrease in intermuscular fat content, can be found in MSTN mutation cattle compared to wild-type cattle [37,38]. Notably, the fatty acid contents of the meat from MSTN mutation cattle exhibit lower saturated fatty acid content [39,40].

In our study, there were significant changes in the content and proportion of fatty acids, particularly polyunsaturated fatty acids, in the livers of MSTN mutation cattle compared to wild-type cattle. Polyunsaturated fatty acids have been shown to affect energy metabolism in liver and fat cells, leading to reduced fat accumulation [35]. Similarly, according to He et al., it was found that reduced MSTN resulted in increased levels of polyunsaturated fatty acids, especially arachidonic acid, in rams [41]. Consequently, the increased content of polyunsaturated fatty acids such as arachidonic acid and linoleic acid in our study may contribute to the observed decrease in fat deposition resulting from MSTN mutation.

Furthermore, in the current research, we found changes in bile acid content and proportion and elevated CA, TCA, and GCA levels in the livers of MT cattle. Consistent with our previous study, we suggest that MSTN mutation promotes primary bile acid synthesis [42]. This phenomenon is similar to the decrease in serum triglyceride levels and the change in the content and proportion of the original bile acids caused by direct administration of CA in mice [24]. In addition, according to Zhao et al., more primary bile acids were found in the rumen of cattle with high feed efficiency [43]. The present results also suggest in another way that the reduction of fat deposition caused by MSTN mutation is related to the content and proportion of primary bile acids.

In the gut, we found little difference in total bile acid content between MT and WT cattle, suggesting that the gut does not produce significant bile acid accumulation, despite increased bile acids in the liver. It has been reported that bile acids, especially primary bile acids, accumulate in large quantities in intestines, causing intestinal inflammation and further damage [44,45]. Similarly, according to Lin et al., the proportion of TCA and GCA increased, and the proportion of CA decreased, in the small intestine, especially in the ilea, of cows with higher milk yield [46]. In our results, the CA content in the intestinal bile acid proportion of MT cattle was significantly lower than that of WT cattle, whereas the content of other coupled bile acids and secondary bile acids increased, generally alleviating concerns about intestinal inflammation after MSTN mutation. Studies will be needed to investigate the potential of MSTN to cause gut dysbiosis, due to changes in the bile acid metabolism and the proportions of the intestinal microbiota.

As reported by Xin et al., following MSTN knockdown, the activity of many key enzymes involved in fatty acid β-oxidation and glycolytic processes in cattle was increased, triggering activation of the AMPK signaling pathway, which regulates glucose and lipid metabolism by increasing the AMP/ATP ratio [47]. In this study, we found that MSTN mutation changed the lipid content of the MT cattle livers, and these metabolites are mainly associated with sterol synthesis, bile acid primary metabolism, cytochrome P450, and fatty acids catabolism, as well as other metabolic pathways. MSTN is known to interact with other signaling pathways, such as the insulin-like growth factors (insulin/IGF-1) and AMP-activated protein kinase (AMPK) pathways, which play essential roles in lipid and energy metabolism [11,48]. The MSTN mutation could potentially alter the crosstalk between these pathways, leading to the observed changes in bile acid content and lipid composition.

Bile acid metabolism mainly involves the synthesis, secretion, transport and regulation of bile acids, and involves a large number of catalases, transcription factors, translocation proteins and cell membrane receptors [49,50]. The liver plays a central role in the synthesis and metabolism of bile acids and lipids. MSTN has been shown to regulate key hepatic enzymes involved in lipid and bile acid metabolism, such as SREBP-1 and FXR [51,52]. The MSTN mutation could affect the activity or expression of these enzymes, thereby influencing bile acid and lipid homeostasis. In this study, in order to reveal the effect of MSTN mutation on bile acid metabolism and lipid metabolism, we analyzed the expressions of related genes by qPCR. In MT cattle, the expressions of genes implicated in bile acid synthesis, maturation and secretion, such as CYP7A1, CYP27A1, BAAT, SULTA1, BESP and ABCB4, were significantly up-regulated, suggesting that MSTN mutations promote the processes associated with hepatic bile acid synthesis and secretion. Among them, as we have demonstrated in a previous study, the expression of CYP7A1 and CYP27A1 favored the increase of the bile acid content after MSTN mutation in cattle [25], and in the present study, our results in different samples were consistent with our expectations. Hepatic bile acid metabolism is a complicated regulatory network [53,54]. When bile acids bind to FXR, the expression of SHP is significantly increased, and the activity of LHR-1 or HNF4α is inhibited, leading to the loss of function of the bile acid response element of the CYP7A1 promoter, inhibiting the synthesis of bile acids [21,55]. On the other hand, KLB binds to FGFR4 to form a heterodimeric complex, which is activated in the intestine by the bile acid–FXR–FGF15 signaling pathway and inhibits the transcriptional activity of CYP7A1, thereby inhibiting bile acid synthesis [56,57]. Unfortunately, we have not been able to detect FGF15 protein content or gene expression, although it is expressed in the intestine and transported to the liver via hepatic portal circulation to bind to KLB/FGFR4 [58]. The results showed that the expressions of FXR and SHP in MT cattle were significantly down-regulated, while the expressions of LHR-1 and HNF4α were significantly up-regulated. On the other hand, although we were unable to identify changes in FGF15, we found significantly reduced KLB/FGFR4 expression in the livers of MT cattle. These results support more active liver bile acid metabolism in MT cattle. In addition, we also noticed a significant down-regulation of the expressions of genes involved in lipid synthesis such as PPARα, PPARγ, C/EBPα and SREBP. A similar phenomenon was reported to have been observed in FXR-inhibited mice [20,59]. The expressions of genes related to lipid catabolism and apolipoproteins such as ACACA, APOA1 and APOB were significantly up-regulated. These results also suggested that the MSTN mutation is responsible for reduced lipid synthesis, enhanced lipid catabolism and improved fatty acid metabolism.

In comparison to monogastric animals, bile acid and lipid metabolism in ruminants have been researched relatively little, especially in livestock with MSTN mutations. Ruminant lipid metabolism differs significantly from that of monogastric animals, primarily due to the unique fermentation processes occurring in the rumen [60]. Bile acid metabolism also varies between ruminants and monogastric animals. In cattle, the primary bile acids produced are cholic acid and chenodeoxycholic acid [61], whereas other species, such as mice and humans, produce muricholic acid in addition to these two primary bile acids [62]. Moreover, the enterohepatic circulation of bile acids is less extensive in ruminants than in monogastric animals [42,63]. These differences must be taken into account when investigating the influence of MSTN mutation on bile acid metabolism in cattle. The sample size was not large enough, and the lack of strong statistical evidence is likewise a limitation of the current research.

In conclusion, further research should focus on validating these hypotheses in cattle to provide a more comprehensive understanding of the relationship between MSTN mutation and bile acid and lipid metabolism in ruminants, and to utilize correlation analysis to explain the relationship between bile acids, fatty acids and MSTN expression. These findings may help to elucidate the altered conditions of the metabolism of bile acids and lipid metabolism in response to the MSTN mutation, and also provide basic information and research ideas for subsequent studies.

## 5. Conclusions

MSTN mutation caused changes in fatty acid content and proportion in the livers of cattle, with an increase in polyunsaturated fatty acids and an increase in primary bile acids. The differential metabolites were enriched in sterol synthesis and primary bile acids synthesis. This study provides new basic data for MSTN mutations in beef cattle breeding. However, the specific mechanism of MSTN mutation regulation in this study needs to be further investigated.

## Data Availability

The data presented in this study are available on request from the corresponding author.

## Supplementary Materials

The following supporting information can be downloaded at: https://www.mdpi.com/article/10.3390/metabo13070836/s1, Table S1: Primers of Real-time qPCR; Table S2: Fatty acids content in the livers of WT and MT cattle; Table S3: Bile acids content in the livers of WT and MT cattle; Table S4: Bile acids content in the ileal contents of WT and MT cattle; Table S5: MRM parameters of bile acids; Table S6: Differing metabolites; Figure S1: Relative expression analysis of MSTN in the liver and muscle.
