# Supplementary material for: The Effect of MSTN Mutation on Bile Acid Metabolism and Lipid Metabolism in Cattle
Source: Metabolites. 2023 Jul 11;13(7):836. doi: 10.3390/metabo13070836 (PMC10384915; doi:10.3390/metabo13070836)
Supplement: Supplementary file 1 [file metabolites-13-00836-s001.zip › Supplementary Materia- revision.pdf]

Table S1 : Primers of Real-time qPCR

Gene	Sequences 5′ to 3′
GAPDH-forward	GTGGCAAAGTGGAGATTGTTG
GAPDH-reverse	CTCCTGGAAGATGGTGATGG
CYP7A1-forward	TCGAACTGGAGCTTGTGGAGAGC
CYP7A1-reverse	CGCAGCCTTGTAACAGCACCAG
CYP27A1-forward	CTGCTGCTGACAAGGCTGATCC
CYP27A1-reverse	CCAGAACGATGCGAGCCACAC
MSTN-forward	GCTGTAACCTTCCCAGAACCA
MSTN-reverse	CAATCAAGCCCAAAATCTCTCC
RXRG-forward	CGCACGATGTGGACGCTCAG
RXRG-reverse	CTCAGTGGCTCGGCCTCTCC
RXRB-forward	GCTGCTGCTGCGTCTTCCTG
RXRB-reverse	GGTGTCGCCGATGAGCTTGAAG
RXRA-forward	CAGCCAGGAAGGTTCGCCAAG
RXRA-reverse	CAGCATCTCCATGAGGAAGGTGTC
LRH-1-forward	GCCATGTCTCAGGTGATCCAAGC
LRH-1-reverse	GGCAAGGCAGCGTGGTTCAG
ACACA-forward	CTGACAGACGGCCAGATCCA
ACACA-reverse	CAGATCCTTGTTGTTGTCCCAGA
APOA1-forward	ACCGTGTATGTGGAAGCAATCAAG
APOA1-reverse	TCCCAGTTGTCCAGGAGTTTCAG
C/EBPα-forward	CCTGGAGTTGTTATGGCGTCTTC
C/EBPα-reverse	CTACGACACTCTCGTGCTGCTTC
APOB-forward	TTGACCAAGCCAGCCCTATAACA
APOB-reverse	CCCAGAGTTCAGAGACCTTCAGAGA
PPARG-forward	TGTGAAGTTCAACGCACTGGAATTA
PPARG-reverse	AGGCTTGCAGCAGATTGTCTTGTA
ACACB-forward	GTTGAATCGAATTGAACCCAAACAC
ACACB-reverse	CCTGGATCCTGCAAAGCAAC
HNF4Aforward	GCCACTGCCTAGTGGGAATTAAAG
HNF4Areverse	TGCCTGCATCCACTCAGAAAC
PPARAforward	GGCTGCTATCATTTGCTGTGGA
PPARAreverse	AGACGTCGTCAGGATGGTTGTTC
SULT2A1forward	GGCACACGTCTCATCAGTTCTCA
SULT2A1reverse	GGGATTTCGGACAAGGTAGATCA
SHP-forward	CTAGCTGAGGTGCAGTGGCTACA
SHP-reverse	CTGGCACGTCAGGGTTGAAG
ABCB4-forward	AATTGTGAGCGCAGCCAAAG
ABCB4-reverse	GATGAGGGCTCGGGCAATA
SLC27A5forward	TTGTCCTTGGAGTCCTCAGTTG

SLC27A5reverse	TCGCCCACGTACTGGATCAC
SREBP-forward	AGCCTGGCAATGTGTGAGAA
SREBP-reverse	ACACAGGAGCAGCTGCAAG
LXRA-forward	GGTTCTTCCGCCGCAGTGTC
LXRA-reverse	CTGTTCCTCCTCTTGCCGCTTC
LXRB-forward	CAGACGCTACAACCACGAGACTG
LXRB-reverse	GGTCGGCTGAGAAGATGTTGATGG
FXR-forward	TCTGTGGAGACCGAGCATCTGG
FXR-reverse	TCTTGGCACTTCCTTCGCATGTAC
xFGFR4-forward	AGCACCGTGGCTGTCAAGA
FGFR4-reverse	AGCACACTCCACGATCACGTA
BSEP-forward	CCGCCAGCATCTTTGAGACA
BSEP-reverse	CAATTTCACCCTTAATTCGATCCAG
KLB-forward	AAAGTCCCTAATTCAATGCTGTCC
KLB-reverse	AACTGAGCATGAGCCAGATTAAAG
BAAT-forward	TGCAGAGCAAGCACAGAACATC
BAAT-reverse	CTGTTTCAGGTGACCCACTCCA
ABCC2-forward	TGACAGCCATCTGGCCATTC
ABCC2-reverse	GGCTGTGGACTTGAGCAACAAATA
ABCB1-forward	GCAGCCCACGTCATCAATATC
ABCB1-reverse	CGTCATTAAATGCCACGTTTCC

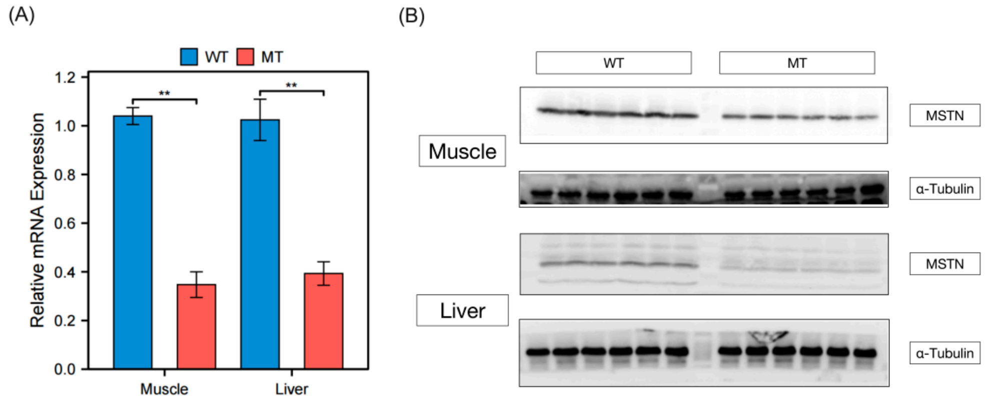

Figure S1 Relative expression analysis of MSTN in the liver and muscle. (A) Expression of MSTN mRNA in the liver and muscle of WT and MT cattle. (B) Expression of MSTN protein in the liver and muscle of WT and MT cattle. ** p < 0.01

Table S2 Fatty acids content in the liver of WT and MT cattle

Class	Abbreviations	Name(μ g/g)	WT (n = 6)	MT (n = 6)	*P* value
MUFA	C15:1	cis-10-Pentadecenoic acid	128.58±4.75	126.59±4.82	0.35301
MUFA	C16:1	Palmitoleic acid	221.88±17.67	211.43±12.12	0.07285
MUFA	C17:1	cis-10-Heptadecenoic acid	136.79±19.12	135.67±21.29	0.87253
MUFA	C18:1n9t	Elaidic acid	216.41±25.69	213.66±26.19	0.77298
MUFA	C18:1n9c	Oleic acid (Octadecenoic acid)	373.98±42.45	512.45±32.58	0.00395
MUFA	C20:1n9	cis-11-Eicosenoic acid	170.26±29.96	170.68±28.02	0.96637
MUFA	C22:1n9	Erucic acid	582.82±140.66	1051.66±556.07	0.01138
MUFA	C24:1n9	Nervonic acid	298.58±33.57	303.49±30.9	0.67739
PUFA	C18:2n6t	Linolelaidic acid	1309.73±263.07	4020.29±349.5	0.00595
PUFA	C18:2n6c	Linoleic acid	259.95±15.14	263.62±19.53	0.59524
PUFA	C18:3n6	y-Linolenic acid	226.68±32.01	355.3±27.01	0.00155
PUFA	C18:3n3	a-Linolenic acid	271.97±27.08	259.58±17.84	0.15458
PUFA	C20:2	cis-11.14-Eicosadienoic acid	158.73±21.6	226.81±18.55	0.00194
PUFA	C20:3n6	cis-8.11.14-Eicosatrienoic acid	1040.6±78.24	2266.59±208.16	0.0036
PUFA	C20:3n3	cis-11,14,17-Eicosatrienoic acid	734.61±66.55	1185.83±78.55	0.005
PUFA	C20:4n6	Arachidonic acid	1403.78±66.49	3028.52±272.3	0.0054
PUFA	C20:5n3	cis-5,8,11,14,17-Eicosapentaenoic acid	616.41±36.15	521.22±40.55	0.008
PUFA	C22:6n3	cis-4,7,10,13,16,19-Docosahexaenoic acid	353.68±35.06	655.91±55.33	0.0011
SFA	C12:0	Lauric acid	243.27±5.16	246.74±3.29	0.24977
SFA	C14:0	Myristic acid	316.49±26.69	345.61±33.97	0.01539
SFA	C15:0	Pentadecanoic acid	155.11±	158.1±	0.7219

			24.08	25.25	4
SFA	C16:0	Palmitic acid (Hexadacanoic acid)	1813.83±97.09	2551.17±182.5	0.0035
SFA	C17:0	Heptadecanoic acid	246.08±32.64	344.96±28.27	0.00484
SFA	C18:0	Stearic acid (Octadecanoic acid)	4635.14±280.61	7627.22±318.3	0.0033

Table S3 Bile acids content in the livers of WT and MT cattle.

Abbreviations	Name (nmol/g)	WT (*n* = 6)	MT (*n* = 6)	p value
CA	Chenodeoxycholic acid	5.15±0.63	8.05±0.31	0.02273
CDCA	Deoxycholic acid	0.13±0.1	0.15±0.1	0.30524
DCA	Lithocholic acid	0.29±0.07	0.4±0.08	0.09975
GCA	Glycocholic acid	156.91±18.54	220.2±14.39	0.03146
GCDCA	Glycochenodeoxycholic acid	3.12±1.47	3.24±0.57	0.43812
GDCA	Glycodeoxycholic acid	27.52±13.51	29.3±11.2	0.30759
GLCA	Cholic acid	0.29±0.07	0.35±0.08	0.79598
GUDCA	Glycolithocholic acid	0.25±0.05	0.16±0.03	0.43958
T-α-MCA	Tauro-α-muricholic acid	0.99±0.3	1.18±0.31	0.20363
TCA	Taurocholic acids	66.55±3.35	184.08±10.59	0.03396
TCDCA	Taurochenodeoxycholic acid	3.68±0.38	3.88±0.34	0.93744
TDCA	Tauroursodeoxycholic acid	24.84±7.96	26.87±4.65	0.88297
TLCA	Taurolithocholic acid	1.08±0.35	0.92±0.23	0.21836

Table S4 Bile acids content in the ileal contents of WT and MT cattle.

Abbreviations	Name (nmol/g)	WT (*n* =6)	MT (*n* =6)	p value
12-ketoDCA	12-ketodeoxycholic acid	13.66±3.67	12.04±3.97	0.74481
ACA	Acetylcholic acid	504.79±67.77	509.25±30.58	0.33286

CA	Cholic acid	10886.86±427.9	2394.1±343.04	0.00137
CDCA	Chenodeoxycholic acid	41.1±10.93	43.23±12.77	0.58211
DCA	Deoxycholic acid	330.99±42.18	347.27±56.38	0.32943
GCA	Glycocholic acid	28097.2±2582.51	29578.4±1645.7	0.82124
GCDCA	Glycochenodeoxycholic acid	1768.23±976.18	2173.02±491.96	0.49786
GDCA	Glycodeoxycholic acid	129.47±27.68	296.31±20.11	0.00308
GLCA	Glycolithocholic acid	80.83±23.92	82.09±28.45	0.97642
GUDCA	Glycoursodeoxycholic Acid	9.92±2.9	8.19±0.39	0.1006
LCA	Lithocholic acid	2.64±0.57	3.32±0.11	0.50133
nutriCA	Nutritional cholic acid	2.05±0.99	1.6±0.25	0.19892
T-α-MCA	Tauro-α-muricholic acid	15.33±2.17	18.09±4.52	0.16045
TCA	Taurocholic acids	25967.26±1452.57	34949.68±2490.93	0.00025
TCDCA	Taurochenodeoxycholic acid	1452.03±511.7	1720.06±574.18	0.57866
TLCA	Taurolithocholic acid	106.71±27.78	118.76±11.15	0.2834

Table S5 MRM parameters of bile acids

No	Analytes	Specie	Quantifier MRM transitions	Qualifier MRM transitions	Retention time (min)
1	T-α-MCA	taurine-conjugated	514.3>80	514.3>124	1.885
2	T-β-MCA	taurine-conjugated	514.3>80	514.3>124	1.944
3	THCA	taurine-conjugated	514.3>80	514.3>124	2.366
4	TUDCA	taurine-conjugated	498.3>80	498.3>124	2.784
5	TCA	taurine-conjugated	514.3>124	514.3>80	2.887
6	GHCA	glycine-conjugated	464.6>74.1	464.6>354.3	2.838
7	GCA	glycine-conjugated	464.3>74.1	464.3>402.4	3.449
8	ω-MCA	unconjugated	407.3>405.3	407.3>387.3	3.461
9	GUDCA	glycine-conjugated	448.3>74.1	448.3>386.3	3.477
10	GHDCA	glycine-conjugated	448.3>74.1	448.3>386.3	3.583
11	α-MCA	unconjugated	407.2>405.3	407.2>387.3	3.73
12	TCDCA	taurine-conjugated	498.3>80	498.3>124	4.066
13	β-MCA	unconjugated	407.4 >407.3	407.3>371.3	4.115
14	HCA	unconjugated	407.3>407.3	407.3>371.3	5.029
15	TDCA	taurine-conjugated	498.3>124	498.3>80	5.417

16	ACA	unconjugated	407.3>361.3	407.3>363.3	5.863
17	CA	unconjugated	407.3>343.3	407.3>289.2	6.007
18	GCDCA	glycine-conjugated	448.3>74.1	448.3>386.3	6.73
19	UDCA	unconjugated	391.3>391.3	391.3>373.5	6.776
20	HDCA	unconjugated	391.3>391.3	391.3>373.3	7.092
21	GDCA	glycine-conjugated	448.3>74.1	448.3>402.3	7.271
22	nutriCA	unconjugated	389.3>389.5	389.3>343.4	7.834
23	12-ketoDCA	unconjugated	389.5>389.5	389.5>343.3	8.033
24	TLCA	taurine-conjugated	482.3>80	482.3>124	8.431
25	CDCA	unconjugated	391.3>391.3	391.3>373.3	8.754
26	DCA	unconjugated	391.3>345.3	391.3>343.3	8.929
27	GLCA	glycine-conjugated	432.3>74.1	432.3>386.3	9.199
28	iso-DCA	unconjugated	391.3>345.3	391.3>327.3	9.934
29	iso-LCA	unconjugated	375.3>375.3	359.3>81.1	10.391
30	LCA	unconjugated	375.3>375.3	359.3>81.1	10.679
